# TNF Signaling Is Required for Castration-Induced Vascular Damage Preceding Prostate Cancer Regression

**DOI:** 10.3390/cancers14246020

**Published:** 2022-12-07

**Authors:** John J. Krolewski, Shalini Singh, Kai Sha, Neha Jaiswal, Steven G. Turowski, Chunliu Pan, Laurie J. Rich, Mukund Seshadri, Kent L. Nastiuk

**Affiliations:** 1 Department of Cancer Genetics & Genomics, Roswell Park Comprehensive Cancer Center, Buffalo, NY 14263, USA; 2 Department of Cell Stress Biology, Roswell Park Comprehensive Cancer Center, Buffalo, NY 14263, USA; 3 Laboratory of Translational Imaging, Center for Oral Oncology, Roswell Park Comprehensive Cancer Center, Buffalo, NY 14263, USA; 4 Department of Urology, Roswell Park Comprehensive Cancer Center, Buffalo, NY 14263, USA

**Keywords:** photoacoustic imaging, tumor hypoxia, contrast-enhanced ultrasound, tumor perfusion, power Doppler, tumor blood flow, endothelial, sTNFR2-Fc, cancer therapy, mouse models

## Abstract

**Simple Summary:**

Androgen deprivation therapy (ADT) is the principal therapy for advanced prostate cancer. ADT controls tumor growth by rapidly altering the prostate tumor microenvironment and subsequently inducing cancer cell death. ADT induces prostate vascular damage and thereby reduces intratumoral blood flow in the rodent and human prostate gland, but the mechanism whereby ADT induces vascular damage has long been elusive. This work describes studies that, for the first time, functionally define TNF as the mediator of castration-induced vascular damage in prostate tumors.

**Abstract:**

The mainstay treatment for locally advanced, recurrent, or metastatic prostate cancer (PrCa) is androgen deprivation therapy (ADT). ADT causes prostate cancers to shrink in volume, or regress, by inducing epithelial tumor cell apoptosis. In normal, non-neoplastic murine prostate, androgen deprivation via castration induces prostate gland regression that is dependent on TNF signaling. In addition to this direct mechanism of action, castration has also been implicated in an indirect mechanism of prostate epithelial cell death, which has been described as vascular regression. The initiating event is endothelial cell apoptosis and/or increased vascular permeability. This subsequently leads to reduced blood flow and perfusion, and then hypoxia, which may enhance epithelial cell apoptosis. Castration-induced vascular regression has been observed in both normal and neoplastic prostates. We used photoacoustic, power Doppler, and contrast-enhanced ultrasound imaging, and CD31 immunohistochemical staining of the microvasculature to assess vascular integrity in the period immediately following castration, enabling us to test the role of TNF signaling in vascular regression. In two mouse models of androgen-responsive prostate cancer, TNF signaling blockade using a soluble TNFR2 ligand trap reversed the functional aspects of vascular regression as well as structural changes in the microvasculature, including reduced vessel wall thickness, cross-sectional area, and vessel perimeter length. These results demonstrate that TNF signaling is required for vascular regression, most likely by inducing endothelial cell apoptosis and increasing vessel permeability. Since TNF is also the critical death receptor ligand for prostate epithelial cells, we propose that TNF is a multi-purpose, comprehensive signal within the prostate cancer microenvironment that mediates prostate cancer regression following androgen deprivation.

## 1. Introduction

Prostate cancer (PrCa) is the second leading cause of cancer-related mortality in American men [1]. While localized PrCa can be cured in many men by surgery or radiation, androgen deprivation therapy (ADT) is the mainstay treatment for locally advanced, recurrent, or metastatic PrCa [2]. Prostate cancer regresses following ADT due to the death of epithelial-derived tumor cells [3]. Indeed, one of the earliest morphological descriptions of apoptotic cell death was in the luminal epithelial cells of the rat prostate gland following castration [4]. Tissue reconstitution experiments by Cunha and colleagues demonstrated that androgen blockade causes prostate cancer regression indirectly via stromal-derived soluble factors that act on the tumor epithelium [5]. For example, we previously demonstrated that stromal-derived tumor necrosis factor (TNF)—the prototypical death receptor ligand—mediates castration-induced regression in normal murine prostates [6]. Specifically, castration-induced regression of the normal prostate is reduced in both Tnfr1- and Tnf-deleted mice as well as mice treated with the TNF ligand trap drug etanercept [6].

Although castration-induced TNF signaling directly triggers epithelial cell apoptotic death in prostate cancers, there is substantial evidence that androgen withdrawal also affects the prostate tumor microvasculature [7,8]. Specifically, castration in rodent models leads to early induction of endothelial cell apoptosis and microvessel leakiness [9], a reduction in blood flow [10,11], and subsequent reduced perfusion of tumor tissue [12], which leads to ischemia and acute hypoxia [13,14,15,16]. A similar process of castration-induced microvasculature damage, caused by paracrine mediators, was observed in primary human xenografts [8]. Therefore, it has been proposed that there is a vascular phase or component to castration-induced regression of prostate cancers during which damage (apoptosis, leakiness) to the tumor microvasculature eventually produces tumor hypoxia. We suggest that the hypoxic state then either initiates or accelerates the necrotic and/or apoptotic death of epithelial tumor cells. The signaling mechanism that mediates ADT-induced vascular damage is unknown, but TNF has been shown to induce both endothelial cell apoptosis and vascular permeability [17,18] and is therefore a candidate for a secreted protein that can function in a paracrine manner to trigger vascular regression.

To test our hypothesis that TNF mediates the vascular component of ADT-induced regression, we monitored the effects of castration on the functional and structural properties of the tumor microvasculature in a c-Myc driven, androgen-sensitive prostate cancer model. We report that castration induced microvessel structural damage, reduced blood flow, reduced perfusion, and reduced oxygenation in the tumor, confirming and extending prior descriptions of vascular events accompanying regression in prostate cancer. Each of these functional and structural vascular alterations induced by castration were reversed by concurrent TNF signaling blockade, demonstrating that endogenous TNF signaling is necessary for castration-induced vascular regression and likely acts at the initiating step of the process, mediating endothelial cell apoptosis and vascular permeability. Combined with our previous studies, our findings suggest a comprehensive role for TNF signaling in ADT-induced regression of prostate cancer. 

## 2. Materials and Methods

### 2.1. Cell Culture

The Myc-CaP cell line was established from the Hi-Myc transgenic mouse prostate [19], which were stably transfected with firefly luciferase under the androgen response element promoter [20]. Myc-CaP/ARE-luc cells were stored as low-passage aliquots, tested for mycoplasma and pathogens, and periodically renewed from frozen stock. Cells were grown in Dulbecco’s Modified Eagle Medium containing glutamine and antibiotics with 10% fetal calf serum. 

### 2.2. Animals

All animal studies were performed in accordance with the National Institute of Health Guidelines for the Care and Use of Laboratory Animals and approved by the Roswell Park Institutional Animal Care and Use Committee (#1308M). Experimental studies were performed using two mouse models of PrCa, subcutaneous allografts of Myc-CaP cells, and an autochthonous, genetically engineered mouse model with prostate-specific Pten deletion [21]. All mice were maintained on a 12-h light and 12-h dark cycle and provided regular chow ad libitum. 

To establish the Myc-CaP tumors, both flanks of male FVB/NCr mice (8–12 weeks old, Charles River Labs) were injected with 5 × 10^5^ Myc-CaP/ARE-luc cells resuspended in equal volumes of RPMI media and Matrigel (Corning, Bedford, MA, USA). Tumor development was monitored by palpation and when evident, quantitated by high-frequency ultrasound (HFUS) imaging, as previously described [22]. Mice were treated and castrated or sham castrated 21 or 24 days after tumor cell injection. The average tumor volume at the time of castration was 382 ± 120 mm^3^.

To produce prostate-specific PTEN loss-induced PrCa-bearing mice [21], male transgenic mice expressing probasin driven Cre recombinase (PB-cre4; obtained from NCI) were crossed with female floxed Pten mouse (Pten^LoxP/LoxP^; obtained from JAX). The resulting pups were individually identified by implantation of a p-Chip (Pharmaseq, Monmouth Junction, NJ, USA) and genotyped for the Cre transgene and LoxP containing Pten alleles. At puberty, the probasin promoter is activated in prostate secretory epithelium, causing epithelial cell-specific deletion of Pten and thereby loss of PTEN activity. Prostate adenocarcinomas form with complete penetrance between 3 and 7 months. The animals were therefore monitored for tumor development using HFUS imaging beginning at 12 weeks. Tumor-bearing animals were enrolled for experimental manipulation when tumor size was between 300 mm^3^ and 700 mm^3^ (mean 436 ± 31 mm^3^).

TNF signaling was blocked by treating mice with etanercept (Amgen, Thousand Oaks, CA, USA), using a regimen previously demonstrated to block castration-induced regression of the normal prostate gland [6]. Etanercept is the soluble extracellular portion of TNFR2 coupled to the IgG Fc domain (sTNFR2-Fc). At three days and one day prior to castration, tumor-bearing mice were treated with sTNFR2-Fc by intraperitoneal (ip) injection (4 mg/kg). Soluble TNF (PeproTech, Rocky Hill, NJ, USA) was injected (27 μg/kg) ip at the time of castration, at a dose sufficient to restore castration-induced prostate regression in TNF-deficient mice [6]. Control mice received PBS on the same schedule. Mice were surgically castrated on day zero, as previously described [23]. Briefly, animals were anesthetized using 2.5% isoflurane (Benson Medical Industries, Markham, ON, Canada) and maintained on 1% isoflurane in oxygen. Testes were removed via bilateral scrotal incision, the blood vessels and vas deferens were ligated, and scrotal incisions were closed by suturing (rather than stapling) to enable imaging of the peritoneum. 

### 2.3. Anatomic and Functional Imaging

Ultrasound imaging with co-registered photoacoustic imaging (PAI) and power Doppler, contrast-enhanced ultrasound (CE-US), and B-mode high-resolution HFUS imaging were performed using a 256 element, 21 MHz linear-array transducer (LZ-250) with the Vevo LAZR system (VisualSonics Inc., Toronto, ON, Canada). After mice were anesthetized and depilated, B-mode ultrasound images were acquired from the peritoneum, and 3D reconstructions of the prostate tumors were computed using Amira 3D visualization software (v5.4.5, FEI Visualization Sciences Group) [22]. For power Doppler sonography, the parameters used for acquisition were: operating frequency, 16 MHz; pulse repetition frequency, 2 kHz; Doppler gain, 40; depth, 20.00 mm; width, 23.04 mm; clutter/wall filter, medium. To enable accurate comparison of the power Doppler data, the relative change in power Doppler signal was calculated for the 3D ROI covering the entire tumor. Multispectral PAI was performed to obtain measurements of oxygen saturation (sO_2_). The PAI parameters used were: operating frequency, 21 MHz; depth, 23.00 mm; width, 23.04 mm; wavelength, 750/850 nm; total hemoglobin concentration threshold (Hbt), 20 arbitrary units; acquisition mode, sO_2_/Hbt. The photoacoustic gain was kept at 43 dB and dynamic range at 20 dB for all studies. PAI-based measurements of oxygen saturation were calculated using the two-wavelength approach (750/850 nm), as previously described [24,25], and the signal was measured for the 3D ROI covering the entire tumor. Nonlinear contrast mode imaging was performed to detect the presence of Vevo MicroMarker contrast agent (VisualSonics, Toronto, ON, Canada). The contrast agent consisted of phospholipid shell microbubbles filled with nitrogen and perfluorobutane (2.3–2.9 μm in diameter). A bolus injection of the contrast agent (1 × 10^8^ microbubbles) was administered via tail vein injection using a 25-gauge needle. Images were acquired using the following parameters: operating frequency, 18 MHz; depth, 20.00 mm; width, 23.04 mm; with 35 dB contrast gain, gate size 6. Nonlinear detection of the contrast signal was performed in 3D by moving the transducer through the volume of the tumor at a step size of 0.152 mm. Multimodal imaging datasets were processed offline using VEVO CQ software (v1.4), utilizing manually drawn tumor regions with perfusion parameters derived from the intratumoral signal intensity time curves for the 3D ROI covering the entire tumor. All imaging datasets were analyzed using Vevo LAB (v.1.7.2) workstation software.

### 2.4. Immunohistochemistry 

Mice were sacrificed 24 h after castration and tumors were collected for histology. Tumors were fixed in formalin-free immunohistochemistry zinc fixative (BD Pharmingen, San Diego, CA, USA) and 4 µm paraffin sections were obtained. Vessels were identified by immunohistochemical staining for CD31 (rat anti-mouse CD31, clone MEC13.3, #550274, BD Pharmingen) using an autostainer (Agilent/DAKO, Carpinteria, CA, USA), as previously reported [12]. Images were digitized using the ScanScope XT system (v12.4.6). The entire tumor area was delineated using Aperio ImageScope v11 software and vasculature integrity was analyzed using the default parameters of the automated microvessel analysis algorithm v1.1 (Aperio Technologies, Vista, CA, USA).

### 2.5. Statistical Analysis

Tumor volume and ELISA TNF protein levels were compared using one-way ANOVA and Dunnett’s test post hoc. Saturated oxygenation, vascularity, and perfusion were analyzed using two-way ANOVA employing Tukey’s honestly significant difference test post hoc to compare treatments over time. ANOVA two-tailed *p*-values < 0.05 were considered significant, and, if reached, post-hoc testing was performed. Unpaired Student’s *t*-test was employed for single-treatment comparisons of histological parameters, and paired Student’s *t*-test was used for single treatments analyzed using pre- and post-imaging. Two-tailed *p*-values < 0.05 were considered significant. All statistical analyses were performed using JMP Pro 11.0 software (SAS).

### 2.6. Data and Materials Availability 

The data generated in this study are available within the article and its Supplementary Data files. 

## 3. Results

To determine if TNF signaling plays a role in the vascular events accompanying castration-induced regression of prostate cancer, we employed a subcutaneously implantable Myc-CaP tumor model. The cell line used in this allograft model was derived from a prostate cancer that developed in Hi-MYC mice [19,26] that express the c-MYC oncogene in the prostate under the control of an androgen-regulated promoter (ARR_2_/probasin-*Myc*). Since c-MYC amplification or overexpression is frequently observed in human prostate cancers [27], this model is relevant to human prostate cancers. We recently studied castration-induced vascular changes in Myc-CaP allografts implanted in mice as part of a study evaluating vascular targeting as a therapeutic strategy for treating prostate cancer [12]. In that report, we documented some of the vascular changes that have been previously described in castration-induced vascular regression [7]. In particular, one day following castration, we observed a reduction in tissue perfusion. Importantly, the effect was similar in tumors implanted subcutaneously and orthotopically, allowing us to reliably use the subcutaneous model in the present study, since signals from some of the tissue imaging modalities we employ to assess vascular function are attenuated by tissue, making it difficult to make measurements on internal organs such as the prostate.

Myc-CaP tumors were implanted subcutaneously and tumor-bearing mice imaged by high-frequency ultrasound (HFUS) to monitor tumor size. Once tumors reached approximately 350–400 mm^3^, mice were castrated and one day later (before there was a significant reduction in tumor volume, Supplementary Figure S1A), we measured changes in each step of the ordered sequence that defines the vascular contribution to tumor regression: (i) microvasculature damage; (ii) reduced blood flow; (iii) reduced tissue perfusion; and (iv) tissue hypoxia. To specifically test the role of TNF signaling, experiments were performed in the presence or absence of sTNFR2-Fc—a soluble form of the TNFR2 extracellular domain fused to the immunoglobulin Fc protein—that binds TNF and prevents signaling in the target cell. 

### 3.1. TNF Is Necessary for Castration-Induced Microvasculature Damage in Myc-CaP Allografts

**Figure 1:**
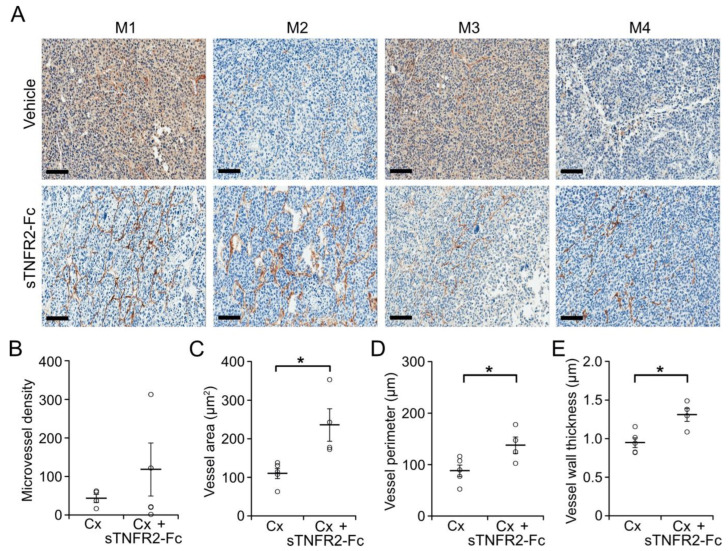
Castration-induced vascular damage is reversed by TNF signaling blockade. (**A**) Representative sections of CD31 immunoreactivity in Myc-CaP prostate tumors from four mice (M1–M4) castrated (upper panels) or castrated and treated with sTNFR2-Fc (lower panels). Scale bars are 100 µm. (**B**) Microvessel density (vessels per square millimeter). (**C**) Vessel cross-sectional area, in µm^2^; (**D**) Vessel perimeter length, in µm; (**E**) Vessel wall thickness, in µm. (**B**–**E**) Large bars, means of measures from the tumors of five vehicle-treated castrated mice and from four sTNFR2-Fc-treated castrated mice (open circles), small bars = SEM. * *p* < 0.05.

To test the role of TNF signaling in vessel structural changes, mice bearing Myc-CaP tumors were treated with vehicle or sTNFR2-Fc, followed by castration. Tumors were excised and formalin-fixed one day after castration, when active androgens were reduced at least 20-fold [28]. The microvasculature network was visualized using CD31 immuno-histochemical staining of tissue sections (Figure 1A) and then analyzed using an automated algorithm that determined microvessel density and related parameters. We previously reported that castration decreased the number of vessels in both subcutaneous and orthotopic Myc-CaP allografts by ~30% after one day [12]. sTNFR2-Fc treatment of castrated mice increased microvessel density in two of four tumors from mice (Figure 1B). In addition, the intratumoral area occupied by vessels, the vessel perimeter, and the thickness of the vessel walls were increased by sTNFR2-Fc treatment in all four tumors examined (Figure 1C–E). Taken together, these data were consistent with TNF mediating the vascular structural damage induced by castration in prostate tumors.

### 3.2. Castration-Induced Reduction in Blood Flow and Perfusion Is Prevented by Blocking TNF Signaling

**Figure 2:**
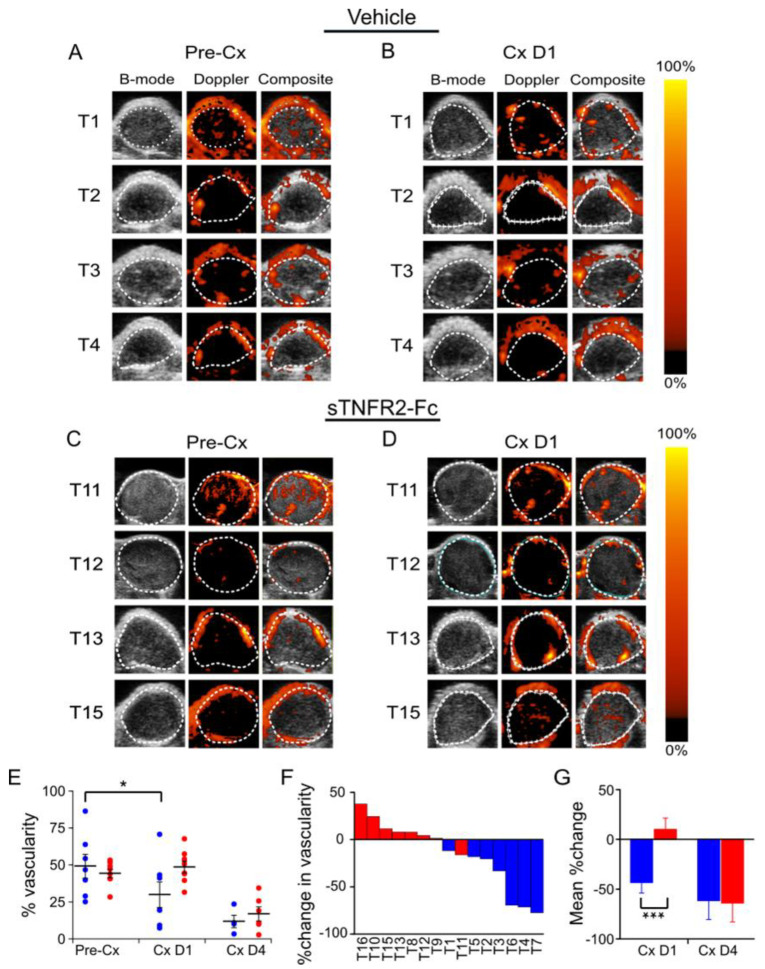
Castration-induced reduction of intratumoral blood flow in Myc-CaP tumor was dependent on TNF signaling. (**A**) Power Doppler (PD) images of subcutaneous Myc-CaP tumors pre-castration (Pre-Cx) of mice treated with PBS (Vehicle), tumors 1–4 (of 8 evaluable tumors). Left to right: Gray-scale ultrasound image (B-mode); PD pseudo-colored image to illustrate blood flow level (Doppler); Composite of PD image overlaid on the B-mode image (Composite). (**B**) PD images of tumors in panel A, one day after castration (Cx D1). (**C**) PD images of a second set of subcutaneous Myc-CaP tumors pre-castration of mice treated with sTNFR2-Fc, tumors 11, 12, 13, 15 (of 8 evaluable tumors). (**D**) PD images of tumors in panel C, one day after castration. (**E**) Mean PD signal (% vascularity, large bars) pre-castration, and at one and four days after castration from tumors in vehicle-treated (blue, D1 n = 8, D4 n = 7) and sTNFR2-Fc-treated mice (red, D1, D4 n = 8), PD signal of each tumor indicated by closed circles. (**F**) Waterfall plot of % change in vascularity in individual tumors at one day after castration. (**G**) Mean % change in paired measures of vascularity pre-castration versus D1 or D4 after castration. Columns and large bars are means and small bars are SEM. * *p* < 0.05, *** *p* < 0.001.

Next, we used power Doppler imaging to quantitate the intensity of intratumoral blood flow. Doppler imaging uses high-frequency sound waves to visualize blood flow magnitude [29]. Power Doppler imaging has increased sensitivity versus color Doppler imaging, allowing more complete vessel function imaging by integrating speed and directional information [30,31]. Castration reduced the blood flow inside tumors (Figure 2A,B), but blood flow was not changed by castration when TNF signaling was blocked using sTNFR2-Fc (Figure 2C,D). While the castration-induced decrease in overall tumor blood flow was TNF-dependent one day after castration (Figure 2E, individual tumor changes at one day after castration are shown in Figure 2F), blood flow continued to decline with time. sTNFR2 treatment was not able to rescue the castration-induced reduction in blood flow four days after castration (Figure 2E). Serial measurement of changes in blood flow inside the same tumor confirmed the castration-induced reduction of blood flow was acutely TNF dependent (Figure 2G).

**Figure 3:**
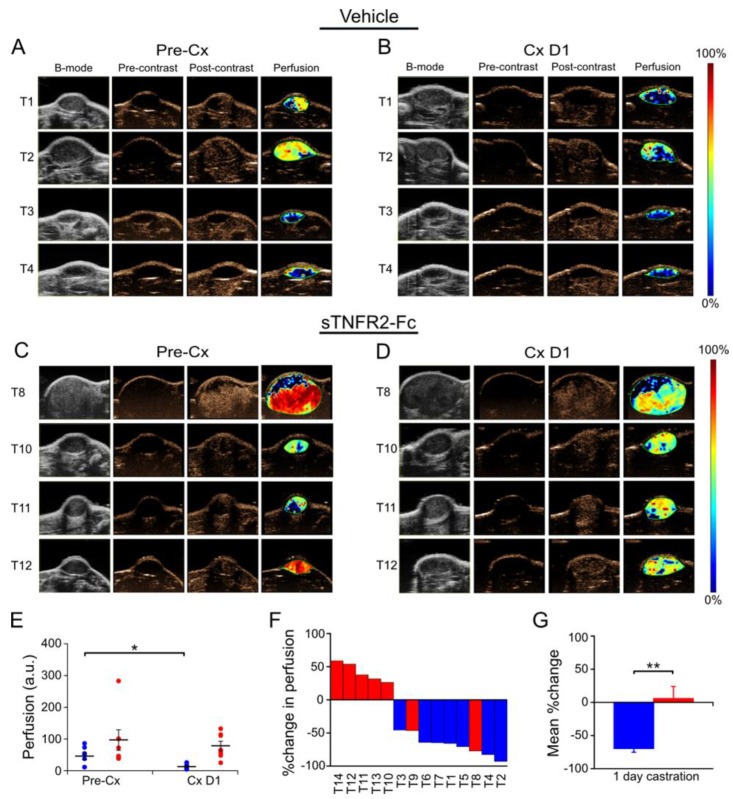
TNF signaling is necessary for castration-induced reduction of perfusion in Myc-CaP tumor. (**A**) Contrast-enhanced ultrasound (CE-US) images of subcutaneous Myc-CaP tumors pre-castration (Pre-Cx) of mice treated with PBS (Vehicle), tumors 1–4 (of 7 evaluable tumors) are shown. Left to right: Gray-scale ultrasound image (B-mode); contrast-mode image prior to contrast agent injection (Pre-contrast); contrast-mode image after injection at the peak enhancement of contrast (Post-contrast); pseudo-colored image of the change in contrast enhancement (Perfusion). (**B**) CE-US images of tumors 1–4 in panel A after one day castration (Cx D1). (**C**) CE-US images of a second set of subcutaneous Myc-CaP tumors pre-castration of mice treated with sTNFR2-Fc, tumors 8, 10, 11, 12 (of 7 evaluable tumors) are shown. (**D**) CE-US images of tumors in panel C, one day after castration, (**E**) Mean perfusion (large bars) pre-castration and post-castration in tumors in vehicle-treated (blue) and sTNFR2-Fc-treated (red) mice, perfusion signal of each tumor indicated by closed circles. (**F**) Waterfall plot of %change in perfusion in individual tumors (columns). (**G**) Average % change in perfusion pre-castration and post-castration. (**E**,**G**): Columns and large bars are means and small bars are SEM. * *p* < 0.05, ** *p* < 0.01.

To test if the changes in blood flow resulted in reduced tumor perfusion, Myc-CaP allografts were evaluated using contrast-enhanced ultrasound (CE-US) prior to and one day after castration of the host animal. Relative perfusion was determined using contrast agent accumulation in tumor capillaries and quantitated from a maximum intensity projection based on contrast accumulation. Figure 3 shows that castration reduced intratumoral perfusion (Figure 3A,B). However, castration did not reduce intratumoral perfusion in sTNFR2-Fc-pretreated host mice (Figure 3C,D). Tumors in sTNFR2-Fc-pretreated host mice were highly perfused, and this was also not changed by castration (Figure 3E). Castration-induced reduction of individual tumor perfusion was dependent on TNF signaling (blue versus red bars in Figure 3F, mean changes for all tumors shown in Figure 3G).

### 3.3. TNF Is Required for Castration-Induced Hypoxia in Myc-CaP Allografts

**Figure 4:**
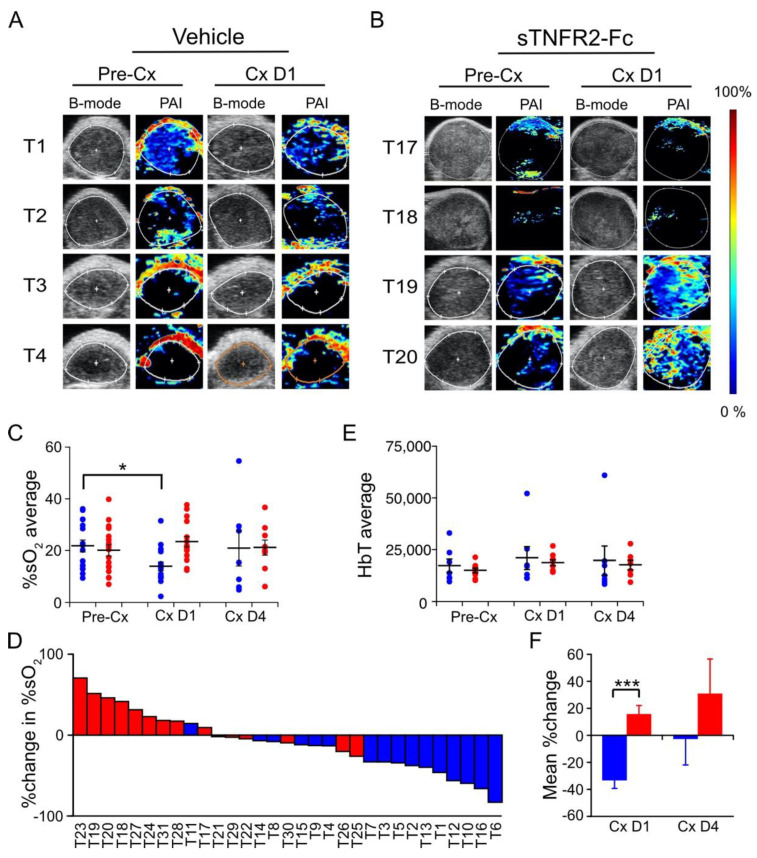
Castration-induced hypoxia in Myc-CaP tumor is reversed by TNF signaling blockade. (**A**,**B**) Photoacoustic images (PAI, pseudo-colored) and ultrasound (B-mode) of Myc-CaP subcutaneous tumors in four tumors from each group, pre- and post-castration from vehicle-treated mice (**A**) or sTNFR2-Fc-treated mice (**B**). (**C**) Mean PAI signal (%sO_2_, large bars) from tumors pre-castration (n = 16), and at one (n = 15) and four (n = 9) days post-castration in vehicle-treated (blue) or in sTNFR2-Fc-treated (red) mice. PAI signal from each tumor indicated by closed circles. (**D**) Waterfall plot of change in %sO_2_ in individual tumors at one day post-castration versus pre-Cx (columns). (**E**) Mean total hemoglobin (large bars) from tumors pre-castration (n = 16), and at one (n = 16), and at four (n = 14) days post-castration in vehicle-treated (blue) or sTNFR2-Fc-treated (red) mice. PAI signal of each tumor indicated by closed circles. (**F**) Mean change in paired measures of %sO_2_ pre-castration versus D1 or D4 post-castration. Mean (columns) and SEM (bars). * *p* < 0.05, *** *p* < 0.001.

Given our observations of reduced blood flow and perfusion, we next sought to determine the level of tissue oxygenation using photoacoustic imaging (PAI) to measure oxygen saturation. PAI can discriminate the absorption spectra of endogenous oxyhemoglobin from deoxyhemoglobin, enabling real-time 3D visualization of the microvasculature and measurement of changes in the percentage of hemoglobin oxygen saturation (%sO_2_) [32]. As in prior experiments, Myc-CaP allograft volumes were measured using HFUS imaging and tumor-bearing mice were pretreated with sTNFR2-Fc (or vehicle) to block TNF signaling, and then castrated. One day after castration, the photoacoustic signal was decreased (Figure 4A) but no castration-induced change in photoacoustic signal was observed if TNF signaling was blocked using sTNFR2-Fc (Figure 4B). Castration induced a relative decrease in intratumoral hemoglobin oxygen saturation (%sO_2_) one day after castration, but not when TNF signaling was blocked in host mice using sTNFR2-Fc (Figure 4C, individual tumor changes shown Figure 4D). The castration-induced hypoxia was not detectable four days after castration. Total intratumoral hemoglobin levels were not changed by either castration or sTNFR2-Fc treatment (Figure 4E). Grouped comparison of paired oxygenation changes in the same tumor revealed that castration-induced hypoxia was TNF dependent (Figure 4F).

### 3.4. TNF Is Required for Castration-Induced Hypoxia in an Autochthonous Prostate Cancer Model

To determine whether castration induces TNF-dependent vascular damage in a more clinically relevant prostate cancer model, we examined vascular change after castration in the endogenously arising PrCa tumors in PbCre4 × Pten^fl/fl^ mice [21]. Tumorigenesis in this model is driven by Pten gene loss in the prostate epithelium. PTEN loss—similar to c-MYC gain of function—is one of the most frequent genetic lesions in both localized and metastatic human PrCa [33] and PTEN loss predicts outcome in patients [34]. Like human PrCa, these tumors grow slowly and regress within weeks after castration [21]. We first determined that TNF signaling did not acutely affect castration-induced regression (at one or four days) or acutely induce intratumoral TNF expression in this PrCa model (Supplemental Figure S2). Thus, these mice provide an excellent model to examine the role of TNF in acute ADT-induced vascular damage. 

**Figure 5:**
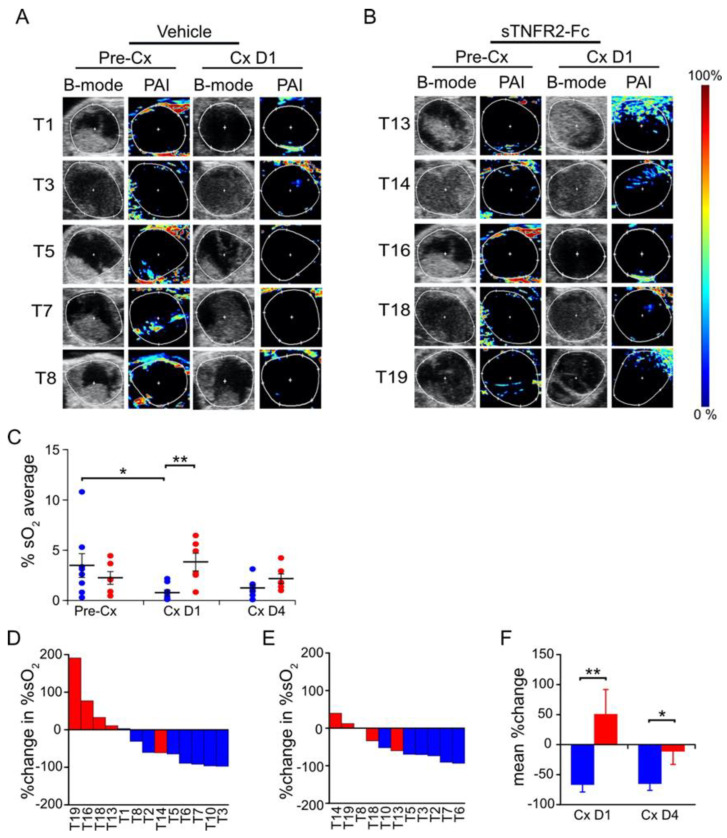
Castration-induced hypoxia is reversed by TNF blockade in prostate tumors of PbCre4 × Ptenfl/fl mice. (**A**,**B**) Photoacoustic images (PAI, pseudo-colored) and ultrasound (B-mode) of five tumors pre- and one day post-castration from vehicle-treated mice (**A**), or sTNFR2-Fc-treated mice (**B**). (**C**) Intra-tumoral mean PAI intensity (%sO_2_ Average) pre-castration, and at one and at four days post-castration in vehicle-treated (n = 8, 8, 7 respectively, blue) or in sTNFR2-Fc-treated (n = 6, 5, 4 respectively, red) mice. (**D**) Waterfall plot of change in intra-tumoral %sO_2_ one day after castration in mice treated with sTNFR2-Fc (red) or vehicle (blue). (**E**) Waterfall plot of change in intra-tumoral %sO_2_ four days after castration in mice treated with sTNFR2-Fc (red) or vehicle (blue). (**F**) Change in paired measures of intra-tumoral %sO_2_ pre-castration versus D1 or D4 after castration. Mean (columns or lines) and SEM (bars). * *p* < 0.05, ** *p* < 0.01.

We tested if castration induced hypoxia in this model and whether the hypoxia was dependent on TNF signaling. Adult PbCre4 × Pten^fl/fl^ mice (tumor volume 575 ± 32 mm^3^) were pretreated with sTNFR2-Fc or vehicle, followed by castration. Tumor hypoxia levels were then assessed serially using PAI. Castration induced a decrease in the average %sO_2_ in the tumors of vehicle-treated mice after one day (Figure 5A,C), but this effect was reduced after four days (Figure 5C). This castration-induced reduction in %sO_2_ was abrogated in the tumors of mice pretreated with sTNFR2-Fc (Figure 5B,C). The observed PA signal (~4% sO_2_) in this C57BL/6 PrCa model was much lower than in the Myc-CaP allografts (~20% sO_2_) in white-colored hosts and in our previous report for a subcutaneous tumor model (~40% sO_2_) in albino hosts [25], likely due to the black pigmentation artifact. Despite lower baseline %sO_2_ levels, the paired change in %sO_2_ (pre- versus post-treatment in the same tumor) was uniformly decreased in vehicle-treated mice at both one and four days after castration (blue, Figure 5D,E). This effect was blocked in most tumors of sTNFR2-Fc-pretreated mice after castration (red, Figure 5D,E). The castration-induced ~50% decrease %sO_2_ (hypoxia) was reversed by sTNFr2-Fc pretreatment (Figure 5F). Power Doppler based measurements of blood flow and CE-US measurement of tumor perfusion were unchanged one or four days after castration in this PbCre4 × Pten^fl/fl^ PrCa model (Supplementary Figure S3, and data not shown). 

### 3.5. TNF Is Necessary but Not Sufficient for Castration-Induced Hypoxia in Myc-CaP Allografts

**Figure 6:**
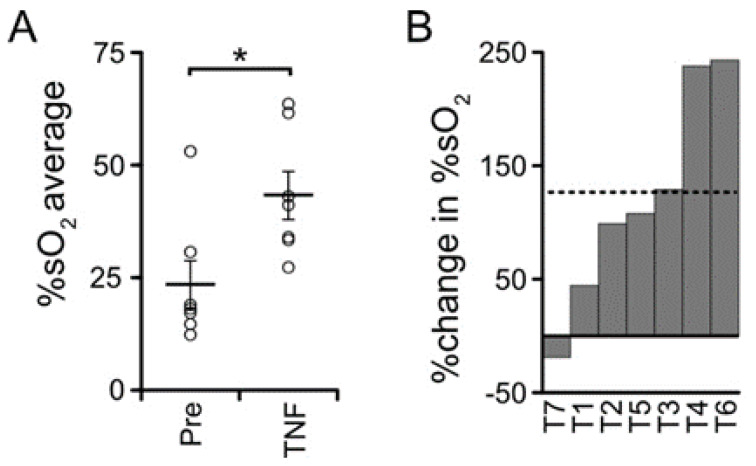
TNF in the absence of castration is insufficient to induce intra-tumoral hypoxia in Myc-CaP tumors. (**A**) Large bars, mean PAI signal (%sO_2_) from tumors pre-castration (n = 7), and at one day following TNF treatment (n = 7). PAI signals from individual tumors (open circles) and SEM (small bars). * *p* < 0.05. (**B**) Waterfall plot of %change in %sO_2_ in individual tumors (columns) after TNF treatment. Mean %change is dashed line.

Finally, since blocking TNF signaling was sufficient to abrogate castration-induced hypoxia, we tested whether soluble TNF was sufficient to induce hypoxia in Myc-CaP tumors. When the Myc-CaP allografts became palpable, tumor volume was measured, PAI was performed, and then host mice were treated with TNF. TNF was administered at a dose sufficient to rescue TNF-mediated prostate regression in TNF-deficient mice [6]. Surprisingly, tumor oxygenation increased one day after TNF injection (Figure 6A). The TNF injection-induced change of %sO_2_ in individual tumors ranged from −20% to 240% (Figure 6B). Moreover, TNF protein levels were unchanged in soluble lysates of whole tumors after castration (Supplementary Figure S1B). These data suggested that in the subcutaneous Myc-CaP model, TNF signaling is necessary, but not sufficient, to trigger castration-induced intratumoral hypoxia. Instead, castration-induced modulation of downstream signaling in vascular cells must be required. 

## 4. Discussion

Previously, we reported that TNF (the prototypical death receptor ligand), but not other death receptor ligands such as FasL or TRAIL, was required for castration-induced regression of the normal murine prostate [6]. This is consistent with the original description of prostate cell death via apoptosis by Kerr et al. [4]. Treatment of normal mice with sTNFR2-Fc did not completely block castration-induced regression, suggesting that other apoptotic mechanisms are required for complete regression of the normal gland [6]. In this report, we employed complementary functional imaging modalities and vessel structural analysis to confirm and extend previous descriptions of the vascular component of regression. This pathological response to androgen deprivation—beginning with endothelial cell apoptosis and increased vessel permeability and culminating in hypoxia [13,14,15,16]—indirectly contributes to prostate cancer regression. In addition to a comprehensive analysis of vascular changes in the subcutaneous Myc-CaP model, we also detected castration-induced hypoxia in a genetically engineered PTEN-deficient prostate cancer model (Figure 5). We were not able to detect reduced blood flow by Power Doppler or reduced perfusion by CE-US in the PTEN-deficient model, perhaps because of limitations in imaging signal penetration for the endogenous prostate relative to the subcutaneous tumors (Figure 2, Figure 3 and Figure 4). 

**Figure 7:**
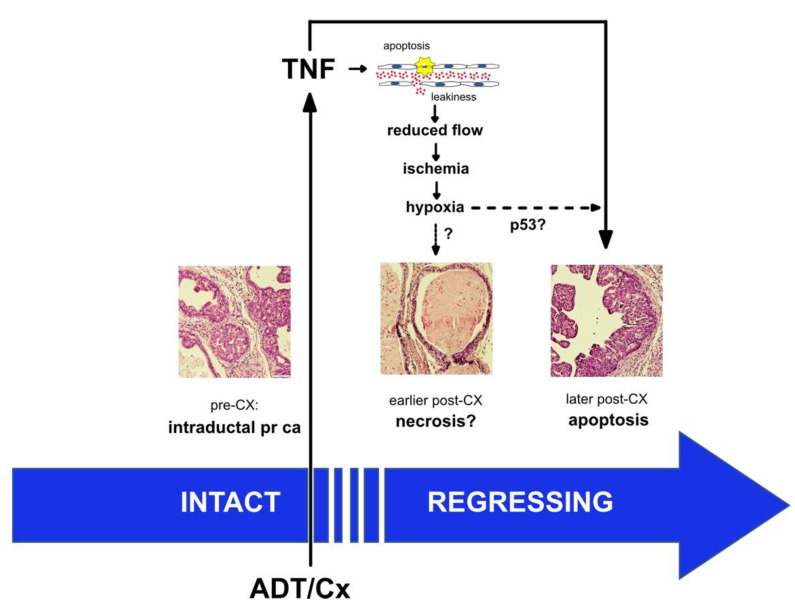
Proposed mechanism for TNF regulation of prostate regression. A proposed mechanism for prostate cancer regression following androgen deprivation therapy is illustrated, focusing on the contribution of events that are mediated by the tumor microvasculature. We propose that one of the earliest events is the activation of TNF signaling in endothelial cells, leading to increased permeability (‘leakiness’) and endothelial cell death. The damage to the endothelium leads to a cascade of vascular changes—reduced blood flow, ischemia due to reduced perfusion, and eventually transient tissue hypoxia—that likely enhances the death of the epithelial component of the tumor. A prediction of the model is that endothelial cell apoptosis precedes epithelial cell apoptosis, which has been noted in the literature (see text). There is limited support for the usual cell death consequence of hypoxia (namely necrosis or necroptosis), but there is a partial requirement for p53 in castration-induced regression, suggesting that p53-hypoxia signaling contributes to death receptor-mediated apoptosis of epithelial tumor cells.

We demonstrated microvessel damage using anti-CD31 immunohistochemistry, reduced blood flow via PD, reduced perfusion via CE-US, and hypoxia via PAI, all of which were inhibited by blocking TNF signaling with sTNFR2-Fc. In agreement with previous reports [8,12,35,36], we found mixed changes in overall intratumoral microvessel density of sTNFR2-Fc-treated host animals after castration; however, we demonstrate TNF is necessary for castration-induced structural vessel damage, including diameter, area, and vessel wall thickness (Figure 1). These events all occur at one day post-castration, prior to the onset of tumor shrinkage (Supplementary Figures S1A and S2A). We have also recently observed that tumor volume in the PTEN-deficient model does not decrease until at least seven days following castration [28]. Despite this delay in tumor regression, intratumoral testosterone and dihydrotestosterone are reduced to near zero within 16 h of castration [28]. It has been shown that apoptosis rates in the endothelium increase prior to those in the epithelium [9], suggesting that endothelial damage occurs earliest. In addition, since we believe that the vascular events in Figure 1, Figure 2, Figure 3 and Figure 4 are sequentially ordered (Figure 7), and since all are inhibited by sTNFR2-Fc, we conclude that TNF acts at the most proximal step. Indeed, there are multiple reports that TNF can induce both endothelial apoptosis and increase vascular permeability [17,18,37,38]. 

Given that TNF signaling is the likely initiating signal for vascular regression, a key question is: what is the molecular trigger that activates TNF signaling in the endothelium? In the normal prostate, we observed an acute increase in levels of TNF mRNA in the stromal compartment approximately 8 h post-castration [6]. In addition, we and others previously showed that c-FLIP is downregulated by castration via the androgen receptor acting on the c-FLIP promoter [39,40]. c-FLIP is a dominant-negative homologue of caspase-8 that acts as a natural inhibitor of death receptor signaling and its sustained expression may play a role in the development of castration-resistant prostate cancer [41]. In the models investigated in this report, TNF levels do not change acutely post-castration (Supplementary Figures S1A and S2A), suggesting that some other component of the TNF signaling cascade is regulated. Since the androgen receptor is only expressed in rodent pericytes, not endothelial cells [42], and pericyte AR regulates perfusion in other organs [43], prostate pericytes may be a key target for androgen regulation of TNF sensitivity via c-FLIP. Other components of the TNF apoptotic signaling network remain potential candidates for activating TNF signaling in the endothelium post-castration. 

Our results also suggest a second key question: how does hypoxia lead to epithelial cancer cell death? One obvious result of tissue hypoxia is necrosis (or perhaps necroptosis). We detected necrosis in some tissue sections from the PTEN-deficient prostate cancer model (Figure 7), but we have not been able to document this on a consistent basis, nor did we observe necrosis in the Myc-CaP model. PTEN-deficient prostate cancers have an intraductal histology, which is observed in only a small fraction of human prostate cancers. Given the multiple layers of cancerous cells lining the ducts in these tumors, PTEN-deficient prostate cancers may be predisposed to hypoxia-induced necrosis. A more likely mechanism, especially in histologically typical prostate cancers, is that hypoxia activates or enhances epithelial apoptosis. Most primary prostate cancers express wild-type functional p53 (Supplementary Figure S4). Hypoxia induces p53 [44], which may then induce apoptosis during prostate cancer regression. Moreover, castration of p53 null mice yields partial regression of the normal prostate [45,46], implying a role for wild-type p53 in castration-induced regression. In the case of DNA damage stress, elevated p53 protein levels are known to induce cell cycle arrest and apoptosis via caspase-9 and Bcl-2 family proteins. However, hypoxia-induced p53 seems to function in a distinct fashion [47], with p53 transcriptionally regulating a distinct set of genes that encode apoptosis regulators [48]. Therefore, p53 induction could be a key molecular signal for hypoxia-induced epithelial apoptosis (Figure 7). Our hypothesis also suggests that patients with mutant p53 prostate cancers will not respond as well to ADT. 

Finally, the TNF-mediated vascular damage caused by ADT may provide an opportunity for prostate cancer therapy. TNF administration induces short-duration vascular disruption in many cancers, transiently enhancing tumor permeability and ultimately reducing blood flow [49,50,51]. TNF therapy induces tumor regression in rodent models [52], but in humans it results in hypotension and other dose-limiting toxicities, thus preventing its effective use as a systemic anti-cancer therapy [53]. Neovasculature-targeted TNF circumvents this toxicity by producing locally high TNF levels and inducing vessel permeability [54], which enhances chemotherapy [55]. Similarly, tumor microenvironment-targeted TNF enhances CD8+ T-cell mediated anti-tumor immunotherapy [56]. We found that ADT, by inducing paracrine TNF signaling, disrupts the tumor vasculature in prostate tumors. However, the ADT-induced vascular damage is not durable, and tumor vascularization increased four weeks after castration [13]. A similar transient effect is observed in human tumors [8]. This suggests that ADT creates a window of vulnerability during which concurrently administered therapies may reduce cancer progression. 

## 5. Conclusions

These results demonstrate that TNF signaling is required for vascular regression, most likely by inducing pericyte apoptosis and increasing vessel permeability. Since TNF is also the critical death receptor ligand for prostate epithelial cells, we propose that TNF is a multi-purpose, comprehensive signal within the prostate cancer microenvironment that mediated prostate cancer regression following androgen deprivation. 

## Data Availability

Data generated or analyzed during the study are available from the corresponding author on request.

## Supplementary Materials

The following supporting information can be downloaded at: https://www.mdpi.com/article/10.3390/cancers14246020/s1. Supplementary Figure S1: Tumor volume and TNF protein levels were not changed post-castration in prostate cancer allografts; Supplemental Figure S2: Tumor volume and TNF protein levels are not changed post-castration in autochthonous prostate cancers; Supplementary Figure S3: Castration does not alter blood flow in prostate tumors of PbCre4 × Ptenfl/fl mice; Supplementary Figure S4: TP53 mutation frequency in primary prostate cancers.
