# Supplementary material for: TNF Signaling Is Required for Castration-Induced Vascular Damage Preceding Prostate Cancer Regression
Source: Cancers (Basel). 2022 Dec 7;14(24):6020. doi: 10.3390/cancers14246020 (PMC9775958; doi:10.3390/cancers14246020)
Supplement: Supplementary file 1 [file cancers-14-06020-s001.zip › cancers-2027538-supplementary.pdf]

656 ## Supplementary Figures

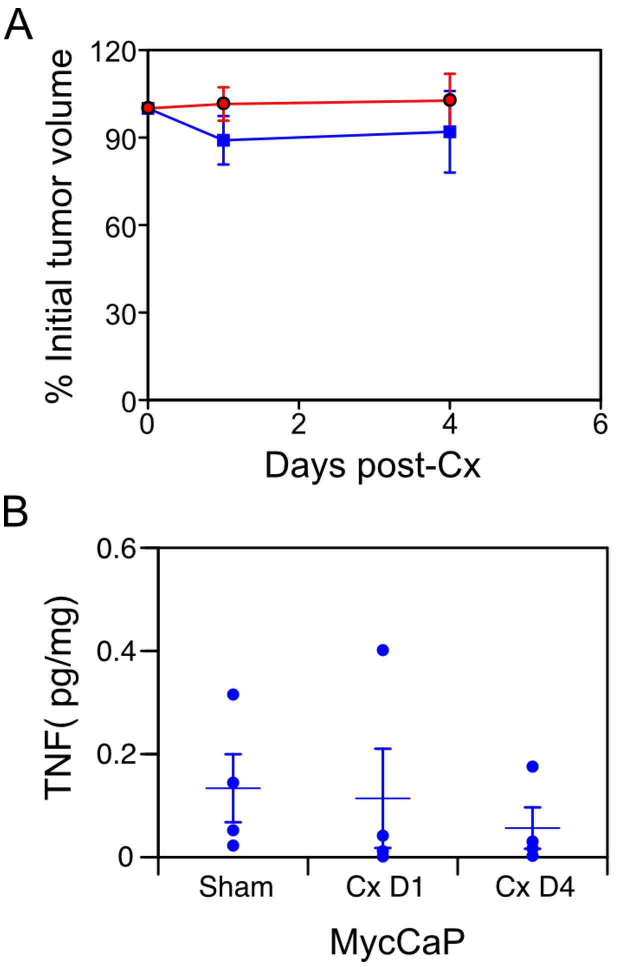

Supplementary Figure S1. Tumor volume and TNF protein levels were not changed post-castration in prostate cancer allografts. A, HFUS determined Myc-CaP tumor volume after castration (normalized to pre-castration volume) in mice treated with vehicle (n=9, blue) or sTNFR2-Fc (n = 7, red). **B,** Tumor tissue was collected from FVB mice bearing Myc-CaP subcutaneous xenografts one and four days after castration (Cx) or from sham-castrated mice. TNF protein levels were measured in solubilized tumor tissue by ELISA (R&D #DY410), pg per mg soluble tumor extract, n = 4 tumors for each group, with individual tumor TNF quantities (circles), and group means (lines) and SEM (bars).

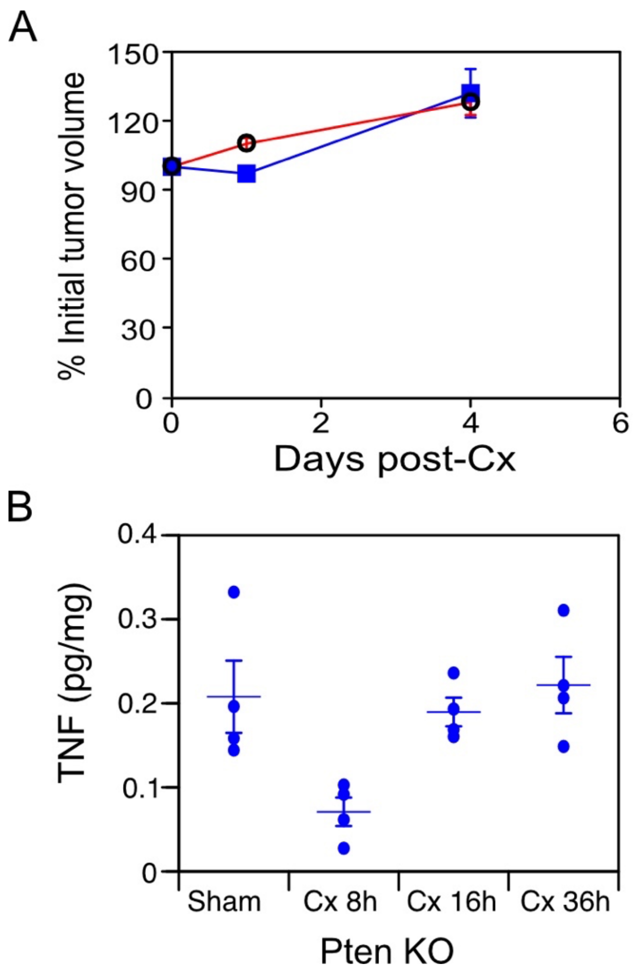

Supplemental Figure S2. Tumor volume and TNF protein levels are not changed post-castration in autochthonous prostate cancers. A, HFUS determined tumor volume post-castration (normalized to pre-castration) in PbCre4 x $Pten^{fl/fl}$ mice treated with vehicle (n=4, blue) or sTNFR2-Fc (n = 4, red). **B,** Tumor tissue was collected from PbCre4 x $Pten^{fl/fl}$ mice at the indicated time (in hours) after castration (Cx) or from sham-castrated mice. TNF protein levels by ELISA, n = 4 tumors for each group, with individual tumor TNF quantities (circles), and group means (lines) and SEM (bars).

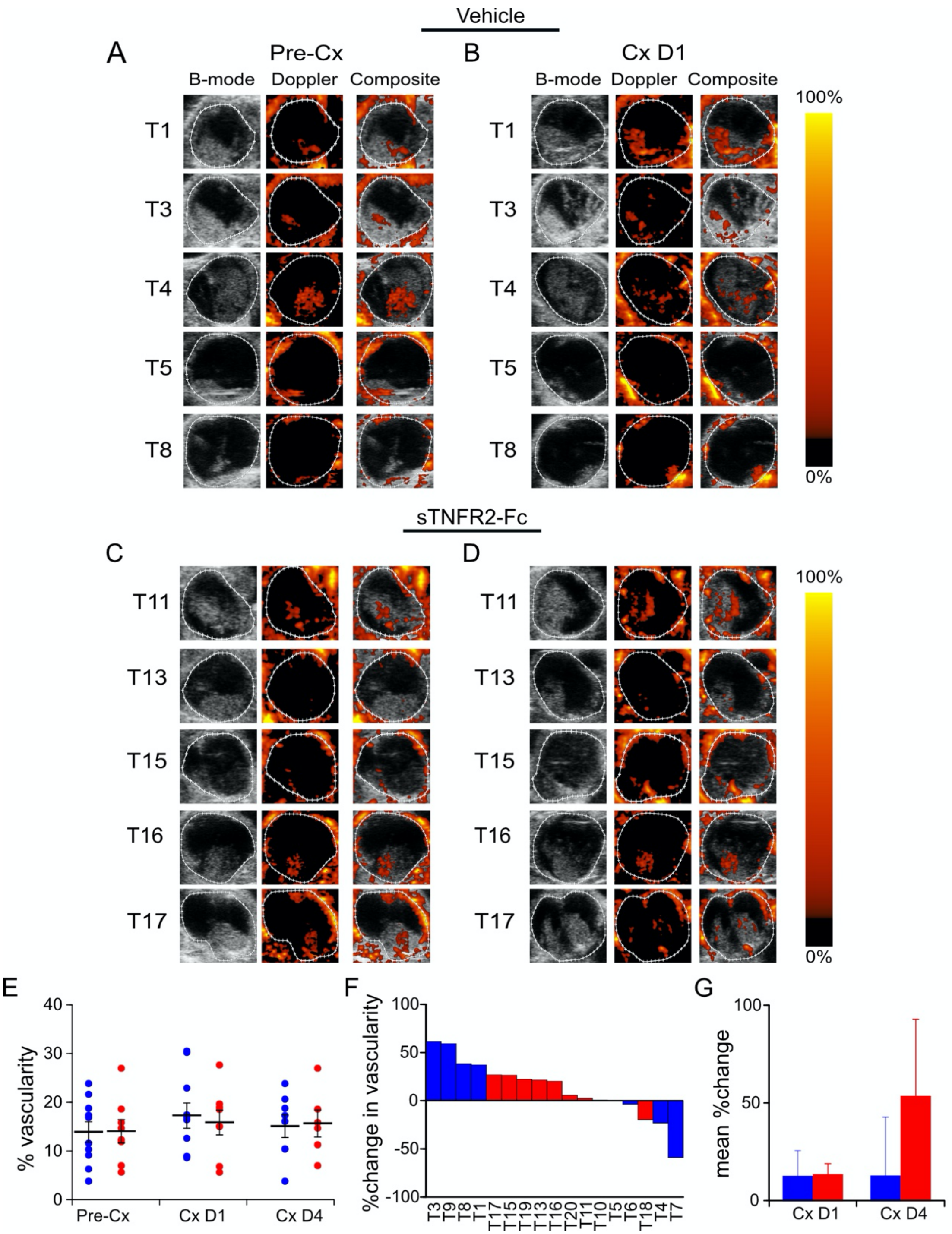

Supplementary Figure S3. Castration does not alter blood flow in prostate tumors of PbCre4 x Ptenfl/fl mice. **A,** Power Doppler (PD) images of subcutaneous PTEN KO tumors pre-castration (Pre-Cx) of mice treated with PBS (Vehicle), 5 tumors (of 10 evaluable tumors) are shown. Left to right: Gray-scale ultrasound image (B-mode); PD pseudo-colored image (Doppler); Composite of pseudo-colored PD image overlaid on the B-mode image (Composite). **B,** PD images of tumors in panel A, one day after castration (Cx D1). **C,** PD images of a second set of PTEN KO tumors pre-castration (Pre-Cx) of mice treated with sTNFR2-Fc, tumors 11, 13, 15, 15 (of 8 evaluable tumors). Images as described in A. **D,** PD images of PTEN tumors one day after castration (Cx D1) of mice treated with sTNFR2-Fc. Images as described in A. **E,** PD signal (% vascularity) from tumors in vehicle treated mice (blue) or tumors in sTNFR2-Fc treated mice (red) pre-castration, and at one and four days after castration. **F,** Waterfall plot of %change in vascularity in individual tumors one day after castration. **G,** Mean %change in paired measures of vascularity pre-castration versus Cx D1 (n = 9 evaluable tumors from vehicle-treated mice; n = 8 from sTNFRII-Fc treated mice) or Cx D4 (n = 7, 5, respectively). Mean (columns) and SEM (bars).

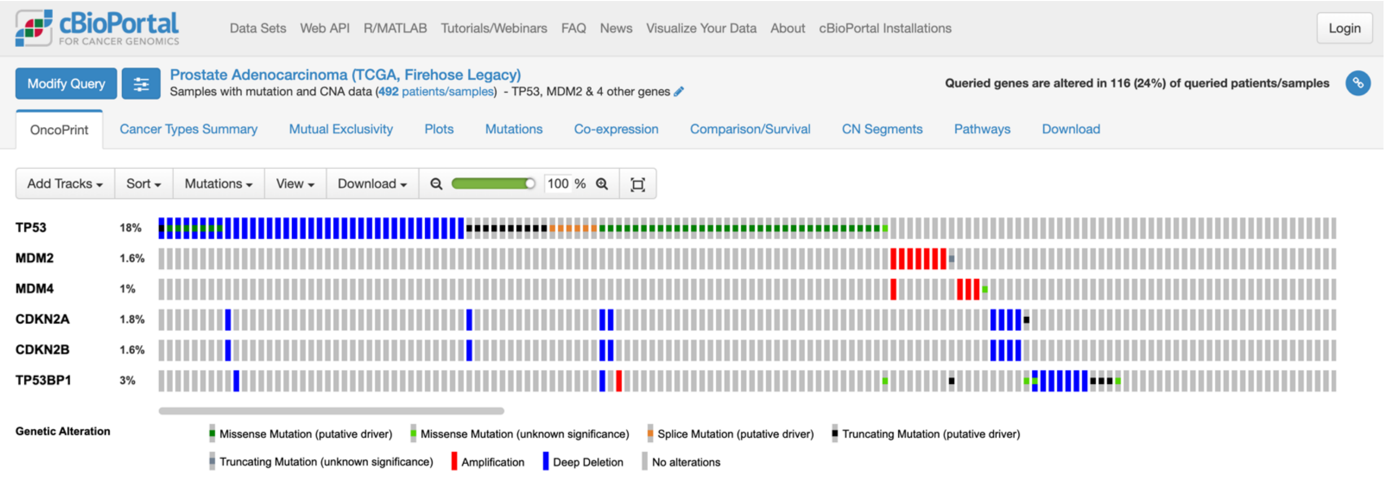

Supplementary Figure S4. TP53 mutation frequency in primary prostate cancers. OncoPrint depiction of cBioPortal analysis of genomic alterations (mutations and copy number alterations) of TP53 and related genes in the TCGA dataset (TCGA, Firehose Legacy) containing 492 primary prostate cancers. The key indicates the color-coding of the alterations. Grey bars have no alterations for the indicated set of 6 genes. Only a portion of the unaltered patient cases are shown.
